# Clinical Case Discussions – a novel, supervised peer-teaching format to promote clinical reasoning in medical students

**DOI:** 10.3205/zma001341

**Published:** 2020-09-15

**Authors:** Nora Koenemann, Benedikt Lenzer, Jan M. Zottmann, Martin R. Fischer, Marc Weidenbusch

**Affiliations:** 1 LMU Munich, University Hospital, Institute for Medical Education, Munich, Germany; 2 Rotkreuzklinikum München, Department of Trauma and Orthopedic Surgery, Munich, Germany; 3 Charité – Universitätsmedizin Berlin, corporate member of Freie Universität Berlin, Humboldt-Universität zu Berlin, and Berlin Institute of Health, Institute of Laboratory Medicine, Clinical Chemistry, and Pathobiochemistry, Berlin, Germany; 4 LMU Munich, University Hospital, Department of Internal Medicine IV, Munich, Germany

**Keywords:** case-based learning, clinical reasoning, peer teaching, curriculum development, undergraduate medical education

## Abstract

**Background:** Clinical reasoning (CR) is a clinical core competence for medical students to acquire. While the necessity for CR teaching has been recognized since the early 20^th^ century, to this day no consensus on how to best educate students in CR exists. Hence, few universities have incorporated dedicated CR teaching formats into their medical curriculum. We propose a novel case-based, peer-taught and physician-supervised collaborative learning format, dubbed “Clinical Case Discussions” (CCDs) to foster CR in medical students.

**Project description: **We present the curricular concept of CCDs and its development according to a six-step approach (problem identification and general needs assessment; targeted needs assessment; goals and objectives; educational strategies; implementation; evaluation and feedback). Our goal is to strengthen the physician roles (CanMEDS/NKLM) and CR competence of medical students. CCDs are offered at our institution as an elective course and students work on real-life, complex medical cases through a structured approach. Over the course of five years we evaluated various aspects of the course and trained student teachers to optimize our course concept according to the feedback of our participants. We also obtained intro and exit self-assessments of CR competence using an established CR questionnaire.

**Results: **We found an unmet need for CR teaching, as medical students in their clinical years view CR as highly important for later practice, but only 50% have ever heard of CR within the curriculum. Acceptance of CCDs was consistently high with over 85% of participants strongly agreeing that they would re-participate in the course and recommend it to a friend. Additionally, we observed significant improvements in CR self-assessments of participants.

**Conclusion: **CCDs are a feasible teaching format to improve students’ CR competence, have a high acceptance and involve students in medical education through peer-teaching.

## Zusammenfassung

**Hintergrund: **Clinical Reasoning (CR) ist eine klinische Kernkompetenz, die Medizinstudierende erwerben müssen. Obwohl schon im frühen 20. Jahrhundert die Notwendigkeit erkannt wurde, CR zu unterrichten, gibt es bis heute keinen Konsens darüber, wie man Studierende am besten in CR ausbildet. Daher haben nur wenige Universitäten dedizierte CR-Lehrformate in ihr medizinisches Curriculum integriert. Wir schlagen mit der sogenannten “Clinical Case Discussion” (CCD) ein neuartiges und fallbasiertes Peer-Teaching-Lehrformat zur Förderung von CR bei Medizinstudierenden vor, das von einem erfahrenen Kliniker supervidiert wird.

**Projektbeschreibung: **Wir stellen das Konzept der CCDs und seine Entwicklung gemäß einer sechsstufigen Herangehensweise vor (Problemidentifizierung und allgemeine Bedarfsanalyse; spezielle Bedarfsanalyse; übergeordnete und spezifische Lernziele; Unterrichtsstrategien und -methoden; Implementierung; Evaluation und Feedback). Unser Ziel ist es, die ärztlichen Rollen (CanMEDS/NKLM) und die CR-Kompetenz Medizinstudierender zu stärken. CCDs werden an unserer Einrichtung als Wahlfach angeboten und die Studierenden arbeiten mittels eines strukturierten Ansatzes an komplexen medizinischen Fällen aus dem echten Leben. Im Laufe von 5 Jahren haben wir verschiedene Aspekte der CCD-Kurse ausgewertet und studentische Lehrende ausgebildet, um unser Kurskonzept auf der Grundlage des Feedbacks der Teilnehmenden zu optimieren. Darüber hinaus wurde deren selbsteingeschätzte CR-Kompetenz mithilfe eines etablierten Fragebogens zur Erfassung von CR jeweils am Anfang und am Ende der CCD-Kurse erfasst.

**Ergebnisse: **Uns zeigte sich ein ungedeckter Bedarf an CR-Unterricht, da Medizinstudierende im klinischen Abschnitt CR zwar als sehr wichtig für die spätere berufliche Praxis erachten, aber nur 50% jemals im Rahmen des Curriculums von CR gehört haben. Die Akzeptanz für die CCDs war durchgehend hoch: 85% der Teilnehmenden stimmten voll zu, dass sie wieder teilnehmen und den Kurs weiterempfehlen würden. Zusätzlich beobachteten wir signifikante Verbesserungen bei den CR-Selbsteinschätzungen der Teilnehmenden.

**Schlussfolgerungen: **CCDs eignen sich als Lehrformat zur Verbesserung der CR-Kompetenz von Studierenden, stoßen auf hohe Akzeptanz und binden die Studierenden über das Peer-Teaching in die medizinische Ausbildung ein.

## 1. Background

There are several factors that influence the quality, efficiency and economic viability of medical care in German hospitals. Clinical reasoning (CR) is a key medical competence to promote patient safety and fosters the economic use of resources (e.g. NKLM [http;://www.nklm.de], CanMEDS [1], PROFILES [2]). Medical students can be taught CR at different points and with various methods throughout their training [3], [4], [5], [6], [7].

Some educators argue that CR is a specific form of scientific reasoning in the medical domain [8], [9]. As such, any curriculum that promotes scientific reasoning should suffice to enable students to reason clinically. To this end, problem-based learning (PBL) [10] is a frequently-used format in medical education [11]. While there is some controversy on the universal definition of PBL [12], most educators would agree that PBL is a student-centered, self-directed small group learning environment, where a problem forms the learning stimulus for the students [10]. Importantly, teachers act as PBL guides facilitating the PBL process rather than experts on the problem matter. Despite its popularity, PBL lacks stringent implementation in many instances [13], [14] and learning objectives are chosen by the students [10]. In contrast, a CR curriculum will have a preset learning objective, namely the improvement of CR competence. Also, there is accumulating evidence for a high context sensitivity of CR competence [15], [16], and probably a combination of knowledge and CR is necessary to build clinical competence [17]. Hence, to foster CR competence we and others favor case-based-learning (CBL) environments [3], [18] which use real-life, complex medical cases and allow for more flexible structures than PBL approaches [10].

Most faculties including our own lack a longitudinal structured CR curriculum with a mix of educational methods and effectively leave CR teaching to circumstance. We therefore aimed to enrich the CR teaching methodology and specifically propose the use of a format we dubbed “Clinical Case Discussions” (CCDs). CCDs are an innovative, supervised peer-teaching format that has now been taught for over five years at the Medical Faculty of LMU Munich. While we recently published the results of a study on the effectiveness of CCDs to promote CR in medical students [19], in this paper we report our real-life experiences with the format, provide instructional material and try to enable interested educators to implement CCDs at their institutions.

## 2. Project description

We describe the curricular development process as well as the implementation of CCDs and share our experiences concerning feasibility and acceptance among participants. We also provide the materials and structure necessary for other faculties to be able to adapt our method and use it for structured teaching of CR. We present our concept based on the six-step approach to Curriculum Development in Medical Education by David E. Kern and colleagues [20]. This concept describes the curricular development in six interconnected steps:

1. Problem identification and general needs assessment
2. Targeted needs assessment
3. Goals and objectives
4. Educational strategies
5. Implementation
6. Evaluation and feedback

### 2.1. Problem identification and general needs assessment

The performance of superfluous diagnostic tests and making medical errors both pose problems within daily medical practice [21]. CR provides physicians with the competence to “think through the various aspects of patient care in order to arrive at a reasonable decision regarding the prevention, diagnosis, or treatment of a clinical problem in a specific patient” [22].

In 1983, Kassirer proposed a bedside teaching approach to teach CR to medical students [23] wherein students who presented cases on wards were given stepwise feedback through an advisor, who is an expert clinician. Active participation plays a crucial role in this method [23] and peer-teaching approaches are particularly known to improve students’ active participation by threshold-lowering [24]. Direct feedback on the reasoning activity of students has also been shown to be important for the further development of reasoning skills [25]. Apart from this method, there is little literature and even less empirical evidence available with respect to case-based, serial-cueing CR teaching formats and their large-scale implementation in medical curricula [3].

#### 2.2. Targeted needs assessment

Our targeted learners are students in various years of the clinical years of medical school who have completed their preclinical training (including basic sciences, anatomy, physiology and biochemistry). By mixing students of various education levels CCDs can be implemented in both traditional as well as hybrid or reformed curricula.

In an online survey conducted in 2017, we assessed medical students’ previous exposure to the term “clinical reasoning” and asked them about their exposure to and learning preference for CR during medical school. Results of the survey with a total of 204 participants (response rate: 8.2% of all students in the clinical semesters) showed that only half of them were familiar with the concept of CR (see table 1 (Tab. 1)). When subsequently presented with a definition of CR, over 90% of participants rated importance of CR for their later work with 8 out of 10 or higher, regardless of their previous exposure to CR. Additionally, more than 50% of participants preferred small group learning for CR teaching.

#### 2.3. Goals and objectives

The main goal of CCDs is the promotion of CR competence. While it is possible to objectively assess CR competence by tests such as the script concordance test [26] or key-feature-based knowledge application tests [27], due to limited resources outside of a research study we used a student self-assessment questionnaire for CR by van Gessel and colleagues [28].

The CCD approach further aims to strengthen several physician roles defined by the CanMEDS and NKLM frameworks [http://www.nklm.de], [1]. The role of *communicator* is strengthened in CCDs by actively encouraging students to share their ideas and thought processes. Becoming a *collaborator* is emphasized by a small group setting and the peer teaching aspect, where there is a low threshold to state own opinions (even though they might be wrong). Through the necessity of making decisions that benefit not only the patient but also by considering other factors such as team-work and efficient use of resources, students learn to act as *professionals* and to be *leaders/managers*. The role of the *scholar* is strengthened by highlighting the importance of self-study, research and staying up to date on developments in the medical field. The peer moderators are not simply giving answers or providing medical facts to the participants, but rather motivate them to find the answers to their questions themselves and share them with the group.

#### 2.4. Educational strategies

CCDs promote case-based collaborative learning, which has been shown to foster CR [29]. The serial-cueing approach embraces the dynamic rather than static nature of the diagnostic process [30]. By applying CR to complex cases, learners train to handle uncertainty through structured en-case-ing the patient [31], i.e. gradually ordering the plethora of signs and symptoms of a patient into functionally related categories and subsequently linking different categories by common underlying pathological processes in a given case. In this regard, the construction of mental frameworks (also called illness scripts) by the learner is an important goal [32]. To ensure a positive learning-atmosphere, we emphasize a “no blame” culture, which forbids ridiculing of false answers or unconventional ideas.

Taken together, the CCD format is built on student autonomy, competence-based learning, and social interaction, all known to promote successful learning [33].

##### 2.4.1. Roles in the CCD

CCDs are a supervised peer-teaching format that comprises four roles:

1. The *discussant*s are medical students in their 3^rd^ through 6^th^ year.
2. The peer-teacher, called the *moderator*, is a medical student and responsible for moderating the discussion. Moderators are previous CCD attendees. They are usually advanced in the course of their studies and are continuously replaced by new generations of moderators as they graduate.
3. For the sake of continuity, quality control and supervision of the student moderator, the course is also attended by a *clinician*. The clinician follows the course of the discussion, and at times uses teachable moments to point out special facts or clinical pearls to the students.
4. One student voluntarily prepares and presents the case. This *presenter* changes every session. By providing teaching on the final diagnosis, the presenter also engages in a peer-teaching activity.

Taken together, the course of a CCD session is primarily determined by the participants’ contributions, while the moderator and clinician ensure effectiveness by limiting discussions on unhelpful or misleading topics. Also, the moderator encourages the participants with guiding questions when the conversation is not flowing.

##### 2.4.2. Case material in the CCD

Cases from the “Case Records” section in the NEJM [https://www.nejm.org/medical-articles/case-records-of-the-massachusetts-general-hospital] suit the CCD approach for four reasons:

1. These cases are real, i.e. nothing is invented or constructed, providing a high level of authenticity.
2. The cases are complex and offer broad differential diagnoses to prompt adequate hypothesis-generation and testing [34].
3. Sufficient data for stepwise disclosure is reported.
4. The “Case Records” include a detailed step-by-step discussion of the case and its peculiarities by an experienced clinician. This facilitates the preparation of the CCD by the moderator as it constitutes externalized expert CR on the case.

Case reports from other medical journals may be suitable for CCDs as well, if they meet the aforementioned criteria.

NEJM case records either emphasize diagnostics or management. While we have used both forms in CCDs, diagnostic cases promote hypothesis-generation more naturally. For selecting cases, we defined “criteria of a good case” (see attachment 1 : supplementary information). The medical specialty involved in a case is secondary to the main goal of providing a systematic approach to cases. We have deployed cases from all medical areas, with a focus on general medicine and neurology (see attachment 1 : supplementary information).

##### 2.4.3. Structure of a CCD session

CCD sessions are divided into three parts:

1. The first part mimics the situation when a physician first encounters a patient. The chief complaint and the history of the present illness, the past medical history, as well as other important information are presented to the students. After presentation of the physical examination results as well as a panel of test results typically performed in the emergency department, the first part of the CCD session ends with the assessment of the patient by means of a short oral summary of the case presented by two or more students. The clinician gives direct feedback on how to improve assessments. The presenter also shows his prepared assessment as a blueprint.
2. The second part of the CCD session directly builds on the assessment. Now guided by the moderator, students develop a prioritized problems list, derive potential differential diagnoses for these problems and order the appropriate tests. Once the students agree that the problems list is complete, the presenter discloses test results of the tests ordered one-by-one. Additional test results are also shown to the students (if these were performed according to the case record). This provides direct feedback to the students as to how appropriate their diagnostic approach to the patient was. Furthermore, it offers students an opportunity to compare standards between their teaching hospital and protocols at the Massachusetts General Hospital. After all but one test result have been disclosed, students are asked to agree on a final presumptive diagnosis and order a last, so-called diagnostic test.
3. In the third and final part of the CCD session, the presenter sums up the CR as described in the original case record before the last diagnostic test is disclosed. By doing so, the students get direct feedback again as to whether they covered all relevant differentials and arrived at the final diagnosis as a result of scrutiny rather than serendipity. After this recapitulation of an expert’s differential diagnosis of the case under discussion, the nature and results of the diagnostic test are disclosed, and the presenter ends the session with some background information on the disease. It is important to understand that the arrival at the final diagnosis is not the primary goal of a CCD session, but rather the demonstration of a structured approach to the case and the generation and subsequent deconstruction of a comprehensive differential diagnosis. Therefore, even a case of a rare disease is applicable if it offers ample differential diagnoses or presents as a more common disease.

#### 2.5. Implementation

The resources necessary for the realization of a CCD are readily available at LMU Munich. The case records are accessible through a university library license. CCD participants also have access to a wide variety of journals and medical literature through the university library. Seminar rooms for CCDs are equipped with a computer and video projector for the case presentation (prepared as a slide show including imaging studies) and a flipchart, used by the moderator for notes (e.g. the problems list). 

Starting in 2014, CCD courses took place as a voluntary elective to all students of the clinical years at LMU Munich. Course language is English, however no minimum proficiency level for entering the CCD was specified. Students can enroll at the beginning of each semester and participate in as many courses as they like, though minimum participation requirements are set to obtain a certificate of participation (70 to 80% course attendance). We advertise the course by holding short presentations at semester opening events, using newsletters, social media and print-posters.

Depending on the staff pool we offered the courses weekly or every other week, splitting the participants into smaller groups. In our experience, participants feel more comfortable in smaller groups, making discussions livelier. Therefore, we set a maximum of twenty-five participants in every discussion group. On average, there were twelve sessions per semester, each session lasting approximately two hours. Moderators and clinicians organize the CCDs voluntarily. Quality standards are ensured by participant evaluation (intro and exit evaluations) as well as informal peer-feedback among moderators and from the clinicians. A structured moderator training takes place once per semester.

#### 2.6. Evaluation and feedback: results from CCD courses 2014-2018

As outlined above, we mainly use participant evaluations for quality control of CCD implementation. The general acceptance of CCD is exemplified by the growing number of participants over the years – from 19 participants in 2014 to 42 participants in 2018, eventually requiring group-splitting to limit group size. A total of 209 students participated in CCDs at LMU Munich from 2014 to 2018. However, as the evaluation form was continually modified over the years, the number of available student answers varies for the items presented here (see table 2 (Tab. 2)).

While 85.14% of participants (63 out of 74) agreed or strongly agreed that they would participate again, 91.89% (68 out of 74) agreed or strongly agreed they would recommend participation to a friend. The overall rating for the CCD format was high (4.54 out of 5).

In respect to the importance of peer-teaching, 89.47% of participants (17 out of 19) agreed or fully agreed that moderators “motivated me to think about the case and to actively participate” and that “the peer teaching concept is a suitable format to learn a clinical case presentation”. The supervising clinician was supporting the group according to 68.42% of participants (13 out of 19).

As CCDs are conducted in English, we wanted to rule out whether English language use was a hindrance to students’ participation. Our evaluation showed that a majority of 72.22% of participants (13 out of 18) had no problems following the course due to the English language, while 55.56% of participants (10 out of 18) indicated they would not prefer to have the course in German if presented with this option.

CCDs are conducted with medical students of different clinical years in one group. When we asked students how they felt about learning collaboratively with students of various clinical years, 93.22% of participants (55 out of 59) agreed or strongly agreed that they profited from working with students in semesters both above and below their own. Concerning the statement that explaining to students from lower semesters or asking questions to students of higher semesters prepared them for their later work in a clinical team, 84.75% of participants (50 out of 59) agreed or strongly agreed. Conversely, 91.53% of participants (54 out of 59) disagreed or fully disagreed that they would have preferred learning with students from only their semester.

As the assessment of objective indicators for successful CR is resource-intensive and could not be performed during routine evaluations, we utilized a CR questionnaire by van Gessel and colleagues [28]. The results of 48 participants, for whom matching forms were available, show significant increases in the overall CR scores from intro (M=3.44; SD=0.58) to exit evaluation (M=3.67; SD=0.35), t(47)=-2.89, p=.006, d=.48. Most profound increases on the single item level were found for the statements “I feel competent applying the clinical reasoning process in patient care” and “I feel competent generating working hypotheses” (see table 3 (Tab. 3)).

## 3. Discussion

We present our five-year real-life experience with a new teaching format, the “Clinical Case Discussions”. CCDs are a case-based, supervised peer-teaching format that adds to the CR teaching methodology.

At most universities there is a wide variety of courses to teach medical students facts, but there are only few classes designed to teach CR competence [35]. For example, we found that at our institution courses such as Medical English and Evidence Based Medicine introduce the concept of CR. However, students want to learn more CR during their clinical core courses. Interestingly, 80% of students from our targeted needs assessment wished for CR to be included in bedside teaching, while about half of the students also opted for classic or PBL seminars. While we also believe that bedside-teaching is suitable to teach CR, a mix of teaching formats may be necessary to teach CR competence effectively [36]. Along with other non-bedside formats [37], [38], CCDs can be used for that purpose. For fostering CR, collaborative learning is superior to individual learning [29]. A specific advantage of CCDs can be seen in the form of discussion, where participants naturally interact.

Students also interact in PBL formats, of course, but CBL formats have been shown to be preferred by students [39], [40]. While reasons underlying student’s preferences for individual teaching formats are manifold, based on learning-theory there are at least two advantages of the CCD format:

1. PBL formats often use a seven-step approach to structure reasoning processes in all educational fields. CCDs are specifically tailored to clinical reasoning by using cases and serial-cueing. The stepwise disclosure of information of a real case in CCD is expected to specifically foster diagnostic reasoning skills.
2. While teachers in PBL act as facilitators and generally do not contribute expert knowledge, the presence of a medical expert during CCDs (i.e. the clinician) creates a learning environment which resembles the “cognitive apprenticeship” approach [41] where an expert (master) supervises a novice (apprentice) engaging in a professional activity. During CCDs, student participants (apprentices) engage in CR (cognitive professional activity) while the clinician (master) supervises and, when necessary, intervenes.

While some authors argue that CR should be taught as early as in the pre-clinical years [4], [42], we deliberately chose to include students of clinical years only. While pre-existing medical knowledge might not be a prerequisite to learn CR [43], in our experience the basic understanding of physiology, biochemistry and anatomy are indispensable to connect individual symptoms or findings and ultimately understand a case. However, when we asked participants about the heterogeneity of prior knowledge, the answers were generally favorable. Currently, more practical experiences are needed to see if CCDs are still feasible when participants differ even more with respect to their prior knowledge (i.e. when pre-clinical students participate).

Despite its importance, today there are limited opportunities for medical students to practice the role of a teacher. Irrespective of the specific professional career, medical graduates need skills to teach effectively [http://www.nklm.de], [1]. Our course implements competence-based teaching by educating students to be peer-teachers. CCD moderators gain first-hand teaching-experience and a chance to grow into their role as lecturers. Additionally, the CCD allows for students to experience the role of a physician-lecturer and participate directly in medical education activities at their university.

However, the data presented here have several important limitations. First, the CCD is an extracurricular course offered at a single institution at this point. Students receive no academic credit and classes are held in the evening, so it is likely that only highly motivated students participate. Interestingly, when we asked participants for both high-school diploma grade (“Abiturnote”) as well as German National Board Exams Step 1 (“1. Abschnitt der Ärztlichen Prüfung”), we did not find any difference to the respective distributions of all medical students at our medical school, suggesting an unbiased sample (at least in respect to these overall indicators of academic performance). Nevertheless, the applicability of CCDs at other medical faculties as well as its acceptance as a regular curricular format remain to be tested.

While we have recently shown effectiveness of CCDs to promote CR competence [19], future studies should investigate in greater detail the roles and cognitive activities in CCDs to gain a better understanding of the learning mechanisms involved. This can be helpful in further developing this teaching format, increasing its effectiveness and efficacy.

Finally, improved CR competence helps medical care providers avoid cognitive biases and consider various factors that affect the development of illnesses [30]. While this might contribute to an increased quality of care for patients, showing such effects has proven to be difficult even for best evidence medical education practices [44]. Hence, the decision for or against the implementation of a relatively resource-intensive teaching format such as CCD in addition to the data presented here will depend on preferences of the local teaching staff, the curriculum, and available resources.

## 4. Conclusion

We propose an interactive, iterative, case-based and supervised peer-teaching format to promote CR in medical students: The Clinical Case Discussion (CCD). In our experience, CCDs are a well-accepted teaching format of growing popularity. In 2015, an award for innovative teaching was presented to the organizers of the CCD courses at LMU Munich. Its international applicability is currently assessed through its use at Jimma University in Ethiopia [45] as well as during international summer schools in cooperation with Weill Cornell Medical School and Washington University of St. Louis, both in the USA. We hope that this article raises interest in the CCD as an educational tool to foster CR in medical students at other medical schools. To promote its use, the online version of this article is supplemented with working materials for the implementation of CCDs.

## Funding

This work was supported by the German Federal Ministry of Education and Research (grant no. 01PB18004C).

## Supplementary information

- CCD rules of conduct
- Criteria of a good case
- List of cases by departments

see attachment 1 .

## Competing interests

The authors declare that they have no competing interests. 

## Supplementary Material

Supplementary information

## Figures and Tables

**Table 1:**
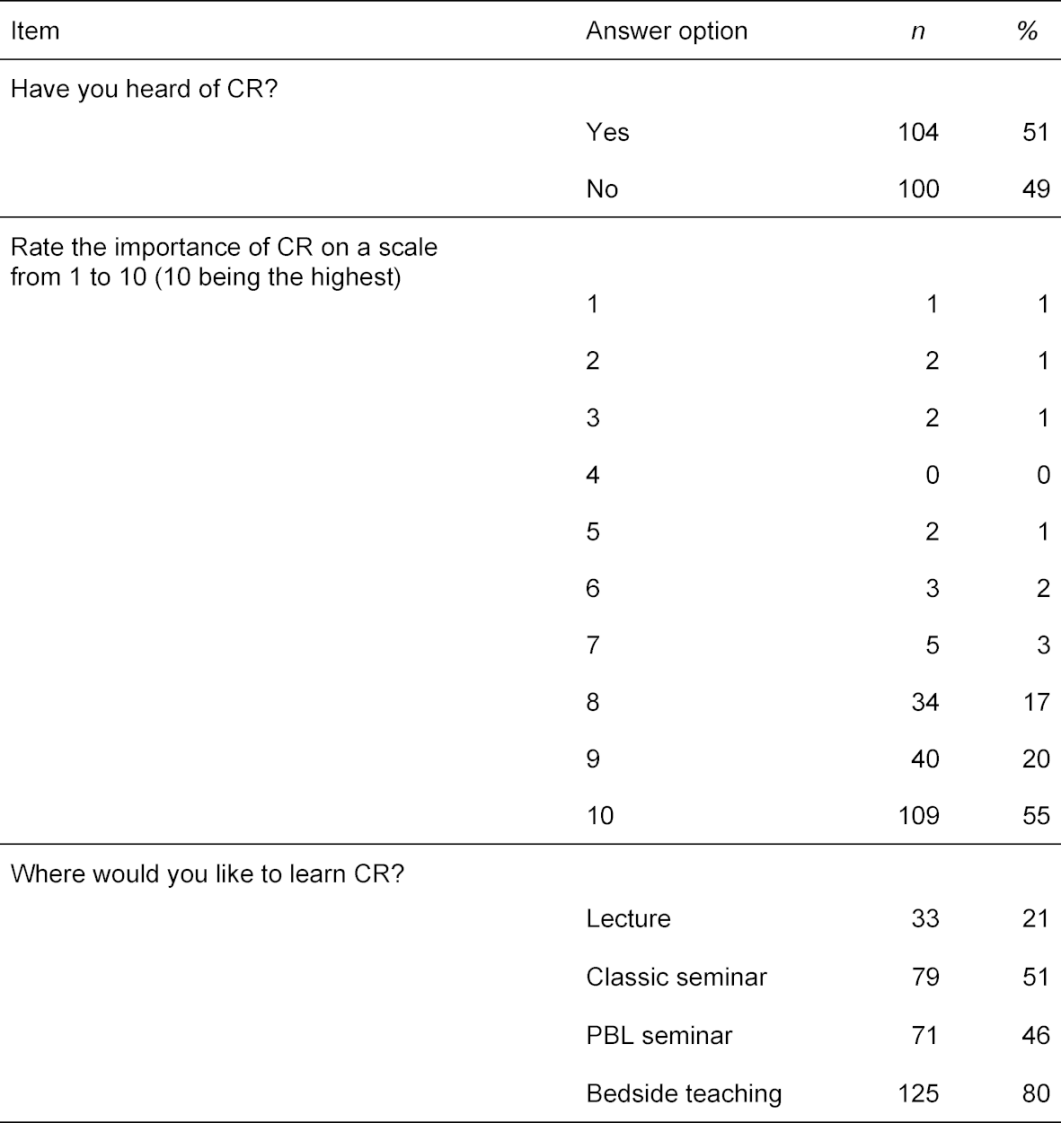
Targeted Needs Assessment Clinical Reasoning

**Table 2:**
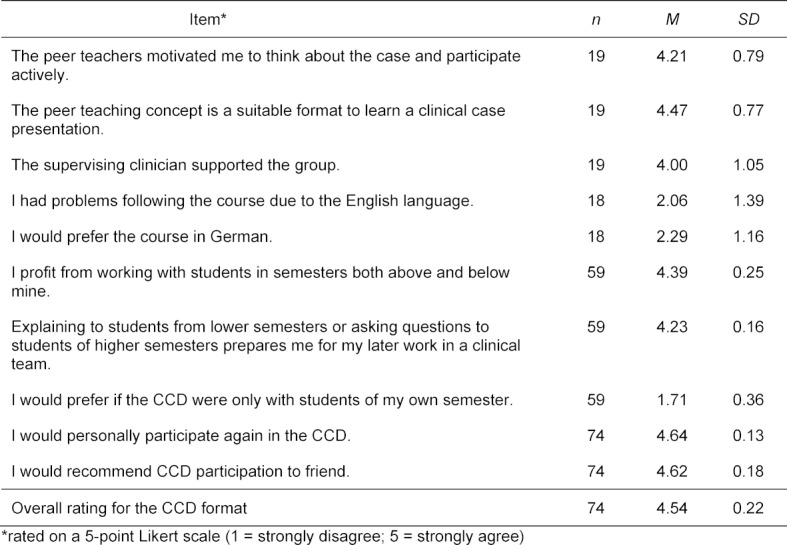
Course evaluation and assessment of contributing factors 2014-2018

**Table 3:**
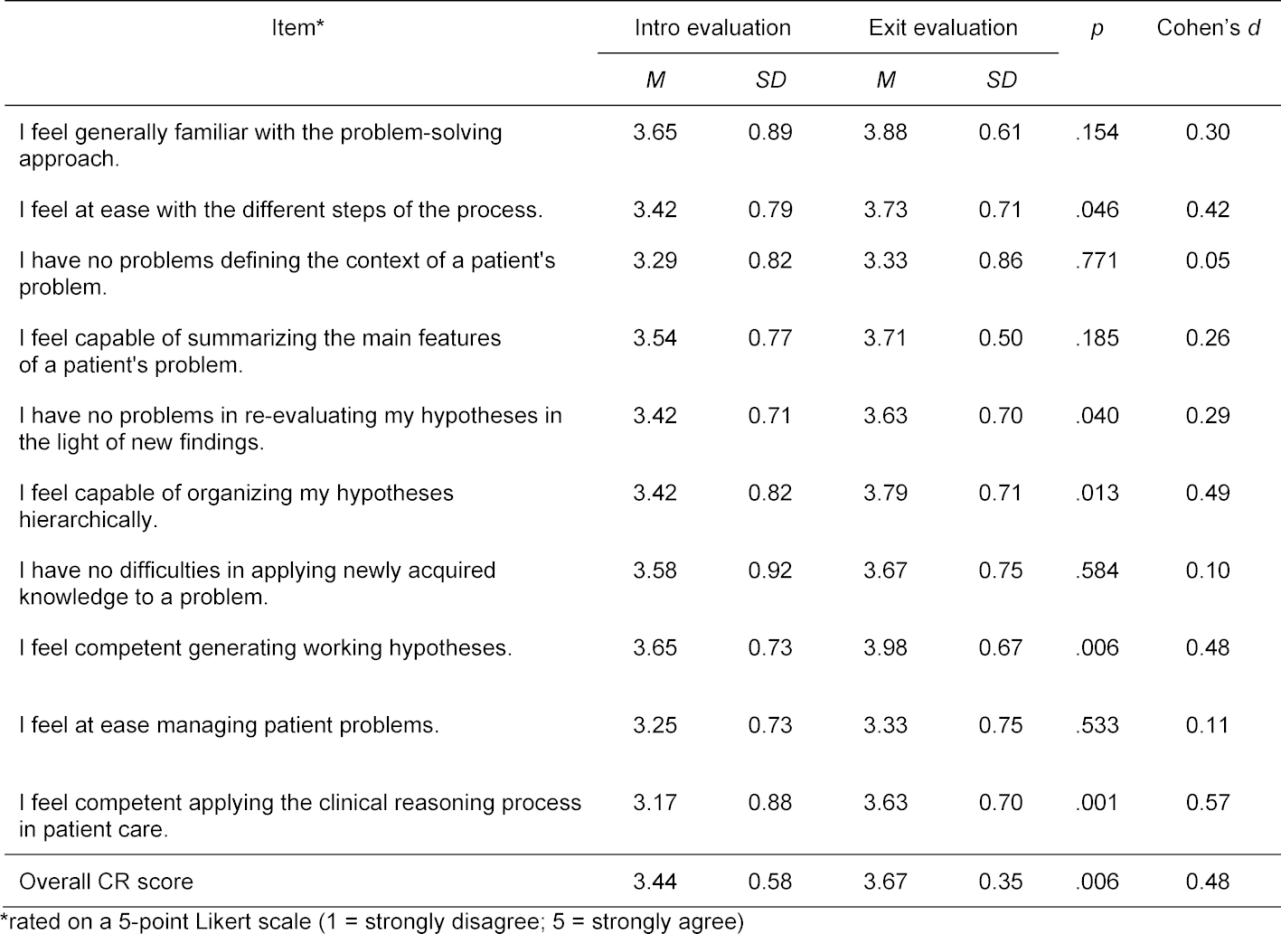
CR scores in the intro and exit evaluation
