# Supplementary material for: Clinical Case Discussions – a novel, supervised peer-teaching format to promote clinical reasoning in medical students
Source: GMS J Med Educ. 2020 Sep 15;37(5):Doc48. doi: 10.3205/zma001341 (PMC7499459; doi:10.3205/zma001341)
Supplement: Supplementary information [file JME-37-48-s-001.pdf]

Supplementary information:

A) CCD rules of conduct

B) Criteria of a good case

C) List of cases by departments

A) CCD rules of conduct (adapted from Univ. Prof. Bernd Gänsbacher)

→ *Small group teaching and learning from each other:*

- Give and take, no passive participation, no spoon-feeding
- The tutor is not supposed to give you all the answers, just guiding questions and hints
- Implementation of a pragmatic - reality based - decision making process
- Learn how to make safe decisions in a setting of limited time and incomplete databases
- During the discussion some questions will not be answered completely – independent study is necessary
- Learn to see the big picture without getting lost in details
- No discussion of unimportant theoretical details for hours
- Differentiate important issues from unimportant issues
- Resources for essential information, easy access (databases, books, internet)
- Use of only the most complete and "peer accepted" information databases (e.g. UpToDate)

→ *Remember:*

- The patient has a right for therapy of first choice (check current guidelines!)
- Know the screening tests for each disease
- In real life as a doctor you will be confronted much more often with rare symptoms of a frequent disease, than with frequent symptoms of a rare disease

→ *Summary:*

- Be active!

Attachment 1 to: *Koenemann N, Lenzer B, Zottmann JM, Fischer MR, Weidenbusch M. Clinical Case Discussions – a novel, supervised peer-teaching format to promote clinical reasoning in medical students.* GMS J Med Educ. 2020;37(5):Doc48. DOI: 10.3205/zma001341

- Participate in the discussion!
- Do not be afraid! (we do not accept making fun of other course participants or unfair criticism)
- English language skills are not important – if you do not know the English word, say it in your mother tongue!

B) Criteria of a good case

The following criteria have proven to make for lively and interesting discussions in the past (not all must apply):

- Final diagnosis has broad differential diagnoses (e.g. at least 3 DDx from 2 different organ systems)
- Rare presentation of common disease or common presentation of a rare disease
- Special patient group (e.g. patients with immunodeficiency, patients with h/o travel)
- Special geographical considerations (e.g. endemic diseases)
- Special periodic consideration (e.g. H1N1 outbreak)
- "Clinical pearl": Case has an interesting aspect/approach that is not commonly known
- If a case is too challenging, students may become frustrated – if it is too easy, students may become bored
- Sufficient diagnostic testing was done (see broad DDx)
- Case is not too long (cases with HPI spanning >10/20yrs can be hard to solve within course time frame)

C) List of cases by departments

Number of cases with the involved departments (according to the information given with each case report from the New England Journal of Medicine):

**Department Involved**	**Number of Cases**
Burn Unit	1
Cardiovascular Medicine	1
Dental Infection and Immunity	1
Emergency Medicine	1
Endocrinology	1
Gynecology	1
Health Care Policy	1
Medical Toxicology	1
Microbiology	1
Nutrition	1
Obstetrics	1
Oral Medicine	1
Pediatric Gastroenterology	1
Pediatric Surgery	1
Physical Medicine and Rehabilitation	1
Reproductive Biology	1
Hematology-Oncology	2
Nephrology	2
Psychiatry	2
Rheumatology, Allergy, Immunology	2
Surgery	3
Gastroenterology	4
Infectious Diseases	4
Cardiology	5
Internal Medicine	5

Attachment 1 to: *Koenemann N, Lenzer B, Zottmann JM, Fischer MR, Weidenbusch M. Clinical Case Discussions – a novel, supervised peer-teaching format to promote clinical reasoning in medical students.* GMS J Med Educ. 2020;37(5):Doc48. DOI: 10.3205/zma001341

Pediatrics	5
Neurology	6
General Medicine	29
Radiology	29
Pathology	33
